# Nucleotide variability and linkage disequilibrium patterns in the porcine *MUC4* gene

**DOI:** 10.1186/1471-2156-13-57

**Published:** 2012-07-13

**Authors:** Ming Yang, Bin Yang, Xueming Yan, Jing Ouyang, Weihong Zeng, Huashui Ai, Jun Ren, Lusheng Huang

**Affiliations:** 1 Key Laboratory for Animal Biotechnology of Jiangxi Province and the Ministry of Agriculture of China, Jiangxi Agricultural University, 330045 Nanchang, China; 2 Department of Biology, Nanchang University of Science and Technology, 330038 Nanchang, China

## Abstract

**Background:**

MUC4 is a type of membrane anchored glycoprotein and serves as the major constituent of mucus that covers epithelial surfaces of many tissues such as trachea, colon and cervix. MUC4 plays important roles in the lubrication and protection of the surface epithelium, cell proliferation and differentiation, immune response, cell adhesion and cancer development. To gain insights into the evolution of the porcine *MUC4* gene, we surveyed the nucleotide variability and linkage disequilibrium (LD) within this gene in Chinese indigenous breeds and Western commercial breeds.

**Results:**

A total of 53 SNPs covering the *MUC4* gene were genotyped on 5 wild boars and 307 domestic pigs representing 11 Chinese breeds and 3 Western breeds. The nucleotide variability, haplotype phylogeny and LD extent of *MUC4* were analyzed in these breeds. Both Chinese and Western breeds had considerable nucleotide diversity at the *MUC4* locus. Western pig breeds like Duroc and Large White have comparable nucleotide diversity as many of Chinese breeds, thus artificial selection for lean pork production have not reduced the genetic variability of *MUC4* in Western commercial breeds. Haplotype phylogeny analyses indicated that *MUC4* had evolved divergently in Chinese and Western pigs. The dendrogram of genetic differentiation between breeds generally reflected demographic history and geographical distribution of these breeds. LD patterns were unexpectedly similar between Chinese and Western breeds, in which LD usually extended less than 20 kb. This is different from the presumed high LD extent (more than 100 kb) in Western commercial breeds. The significant positive Tajima’D, and Fu and Li’s D statistics in a few Chinese and Western breeds implied that *MUC4* might undergo balancing selection in domestic breeds. Nevertheless, we cautioned that the significant statistics could be upward biased by SNP ascertainment process.

**Conclusions:**

Chinese and Western breeds have similar nucleotide diversity but evolve divergently in the *MUC4* region. Western breeds exhibited unusual low LD extent at the *MUC4* locus, reflecting the complexity of nucleotide variability of pig genome. The finding suggests that high density (e.g. 1SNP/10 kb) markers are required to capture the underlying causal variants at such regions.

## Background

The genetic variability of pig genome has been shaped by many evolutionary forces such as domestication and selection. Studying the genetic variability has at least two implications: revealing the evolutionary history of certain breeds; and finding out genomic regions or genes that have been subject to natural or artificial selection, thus helps to dissect the genetic basis of fitness traits.

Analyzing natural and artificial selection pressure on genes of interest can deepen our understanding on these genes. The existing pig breeds have undergone intensive artificial selection during the past decades or centuries, which provide valuable resources for such analysis. For instance, Ojeda *el al.* (2008) conducted a worldwide survey of haplotype variability around *IGF2*, a well-characterized causal gene affecting lean content [1]. They showed a clear selective sweep signature within this gene in Western commercial lean breeds, like Duroc and Pietrain. Similar investigations have also been performed on other genes, such as *FABP4*[2], *SERPINA6*[3] and *PPARD*[4] in pigs. These studies provided interesting information on evolutionary history and functional importance of targeted genes.

Mucins are a family of large membrane-bound or secretory glycoproteins normally produced by epithelial cells of tissues [5]. Mucins play important roles in protecting and lubricating the surface cells, regulating cellular signaling and other biological processes through acting as selective barrier or transmitters of host epithelial cells against external substances or organisms [6,7]. MUC4 is a trans-membrane member of mucin family. Human *MUC4* gene comprises of 26 exons spanning from 65 bp to 22 kb. Exon 2 of human *MUC4* is particularly important as it contains a highly variable tandem repeated sequence that encodes a heavily *O*-glycosylated protein domain forming functional extracellular structures [8]. Abnormal overexpression of *MUC4* is related to progression of several carcinomas [9,10]. Moreover, MUC4 has been shown to be important in lubrication and protection of the surface epithelium, epithelial cell proliferation and differentiation, immune response and cell adhesion [8].

The functional importance of MUC4 in pigs has also been implicated in several studies. *MUC4* expression on uterine epithelial changes during oestrous cycle and early pregnancy, suggesting the relevance of MUC4 in protecting and maintaining the endometrium conditions [11]. *S.typhimurium*-infected pigs show reduced MUC4 expression on the surface of colonic epithelium compared with non-infected pigs, indicated that MUC4 is important in inflammatory response in colonic tissues [12]. Previously, we and other investigators reported *MUC4* polymorphisms that were strongly associated with susceptibility to enterotoxigenic *Escherichia coli* (ETEC) *F4ab/ac* in pigs [13,14], although we recently demonstrated that susceptibility towards ETEC F4ac is governed by the *MUC13* gene proximal to *MUC4* (unpublished data). More recently, a variant in *MUC4* has been evidenced to be significantly associated with prolificacy traits; *MUC4* expression levels in the uterine of high prolific sows are two-fold greater than those in low prolific sows [15].

In this study, we investigated the nucleotide variability, haplotype phylogeny and linkage disequilibrium within the *MUC4* gene in 312 pigs pertaining to 11 Chinese indigenous breeds, 3 Western commercial breeds and 5 wild boars. The findings provide novel insights into evolutionary history and functional importance of the porcine *MUC4* gene.

## Results and discussion

### MUC4 polymorphisms

We identified a total of 90 SNPs by sequencing 32 amplicons (total length ~38.1 kb) of the porcine *MUC4* gene from 4 White Duroc and 4 Chinese Erhualin pigs. We selected 58 SNPs for further genotyping according to their uniformly genomic distribution, reliability and informativness. A final set of 53 SNPs passing the filtering criteria with call rates >90% were used for further analyses. The primer sequences and chromosomal positions of the 53 SNPs are shown in Additional file 1: Table S1 and Additional file 2: Figure S1. A majority of these SNPs (33/53) were segregating in both White Duroc and Erhualian pigs (Additional file 3: Table S2). These SNPs cover a 92-kb region around the *MUC4* gene with an average interval of 1.7 kb. All SNPs were intronic variants except one synonymous mutation on exon 25.

Table 1 presents the number of segregating site (S), the mean number of pairwise differences across loci (π_N_), Tajimas’s D, and Fu and Li’s D statistics in the tested breeds. In Chinese breeds, Tongcheng pigs were the most variable breeds (π_N_ = 0.29), followed by Jiangquhai and Laiwu (π_N_ = 0.26). In comparison, Jinhua (π_N_ = 0.13) and Hang (π_N_ = 0.16) pigs were the least polymorphic breeds. For three Western commercial breeds, White Duroc is the most variable breed (π_N_ = 0.23) while Landrace has the least nucleotide diversity (π_N_ = 0.16). The overall nucleotide variability of Chinese breeds (π_N_ = 0.27) were larger than that in Chinese Wild boars (π_N_ = 0.19). This could be caused by an underrepresented ancestral genetic pool by the 4 Chinese wild boars used in this study. Overall, nucleotide heterozygosities in Chinese breeds (π_N_ = 0.27) was greater than that in Western breeds (π_N_ = 0.23). This is conceivable as the genetic variability of Chinese breeds has been repeatedly evidenced to be greater than that of Western commercial breeds [1-3,16]. Unlike the *IGF2* gene at which intensive selection for growth and lean production has not wiped out genetic variability in Western commercial breeds especially in Duroc and Pietrain [1], the genetic variability around *MUC4* in Duroc was comparable to those Chinese obese breeds like Erhualian (Table 1). Therefore, *MUC4* is obviously not a locus under directional selection for pork production. Although *MUC4* plays important roles in multiple biological processes as mentioned above [8-10,17,18], it probably did not affect growth and fat deposition in pigs. The unusual high nucleotide diversity at *MUC4* e.g., in Duroc and Tongcheng pigs, could be a suggestive signal of balancing selections (discussed below). In a recent genome-wide scan of nucleotide diversity in Western wild boars and commercial breeds, Amaral et al. found high nucleotide diversity in MHC and olfactory receptor genes, indicating an effect of balancing selection [19].

**Table 1:** **Genetic variability around the *MUC4 *gene in Chinese and Western breeds**

**Breed**	**Ecotype**	**N**	**S**	**π**_**N**_	**D**_**T**_	**D**_**FL**_
Chinese local breeds		161	47	0.27	2.69*	2.81**
BamaXiang	South China	16	40	0.25	1.22	1.77*
Erhualian	Lower Yangtse River Basin	32	43	0.19	0.34	1.49
Hang	Central China	10	18	0.16	2.67**	2.19**
Jiangquhai	Lower Yangtse River Basin	12	39	0.26	1.30	1.74*
Jinhua	Central China	11	22	0.13	0.44	-0.82
Laiwu	North China	13	34	0.26	2.10*	2.02**
Rongchang	Southwest China	13	30	0.22	1.75	2.02**
Shaziling	Central China	8	27	0.20	1.17	1.71*
Tongcheng	Central China	10	41	0.29	1.40	1.88**
Yushanhei	Central China	24	27	0.20	2.54*	2.44**
Zangzhu	Plateau	12	29	0.21	1.73	1.99**
Western commercial Breeds		146	37	0.23	2.99**	3.05**
Duroc		32	30	0.22	2.65*	2.36**
White Duroc		16	29	0.23	2.46*	2.33**
Landrace		32	33	0.16	0.79	-0.24
Large White		66	37	0.22	2.17*	2.55**
Wild boars		5	42	0.28	-0.06	0.19
Wild Boars-CN		4	22	0.19	0.87	1.03
ALL		312	53	0.38	4.49***	3.95**

* *P* < 0.05, ** *P* < 0.01, N, number of animals; S, number of segregating sites; *π*_*N*_, mean number of pairwise differences across SNPs; D_T_, Tajima’s D; D_FL_, Fu and Li’s D index. Wild Boars-CN, Chinese wild boars.

### Haplotype reconstruction and phylogenetic analysis

A total of 80 haplotypes were reconstructed using the genotypes of 53 SNPs from all 312 pigs, out of which 139 pigs with 278 reliably inferred haplotypes (*P* > 0.8) were retained for subsequent analyses of haplotype frequency and phylogeny. We focused on 14 major haplotypes with frequencies higher than 0.02 in the 139 pigs. As haplotypes of wild boars were not reliably inferred, ancestral and derived haplotypes can not be firmly determined for the 14 haplotypes. Of these haplotypes, 12 were found in Chinese breeds while only 5 were evidenced in Western breeds. Chinese breeds thus had higher haplotype diversity than Western breeds, which was in agreement with the previous reports [20,21]. Haplotypes 1, 4, 5, 7, 8, 10, 11, 12 and 13 were found exclusively in Chinese indigenous pigs, while haplotypes 6 and 14 were observed only in Western commercial breeds (Table 2). Haplotypes 2, 3 and 9 were presented in both Chinese and Western breeds. For example, haplotypes 2 and 3 were over-dominantly presented in Western commercial breeds, however, they also appeared in Chinese Bama Xiang (0.167 and 0.083) and Laiwu (0.200 and 0.200) pigs (Table 2). The two haplotypes did not have apparent shared segments with Chinese specific haplotypes (data not shown) and was therefore more likely of Western descent. We speculated that they were recently introduced from Western commercial pigs into Bama Xiang and Laiwu pigs. The speculation was supported by our previous finding that 21% of Laiwu pigs carried Western *NR6A1* haplotype, which suggest direct or indirect introgression of Western breeds to Laiwu pigs [22]. In contrast, haplotype 9 looks like a ‘compound’ haplotypes, i.e. mosaics from European and Chinese descent (data not shown). Hence, we can not rule out the possibility that an older introgression of Chinese breeds into Europe pigs happened for the haploptype approximately 200 years ago according to historical records [23].

**Table 2:** **Distribution of 14 main haplotype frequencies in the *MUC4 *gene in corresponding pig populations**

	**N**	**Hap1**	**Hap2**	**Hap3**	**Hap4**	**Hap5**	**Hap6**	**Hap7**	**Hap8**	**Hap9**	**Hap10**	**Hap11**	**Hap12**	**Hap13**	**Hap14**
ALL	278	0.183	0.155	0.115	0.090	0.065	0.043	0.032	0.032	0.029	0.025	0.025	0.025	0.022	0.022
Chinese breeds	182	0.290	0.028	0.017	0.142	0.102	0.000	0.051	0.051	0.011	0.040	0.040	0.040	0.034	0.000
Bama Xiang	12	0.000	0.167	0.083	0.000	0.083	0.000	0.083	0.000	0.000	0.000	0.417	0.000	0.000	0.000
Erhualian	46	0.435	0.000	0.000	0.283	0.065	0.000	0.000	0.000	0.000	0.043	0.000	0.065	0.000	0.000
Hang	18	0.167	0.000	0.000	0.167	0.000	0.000	0.333	0.333	0.000	0.000	0.000	0.000	0.000	0.000
Jiangquhai	22	0.364	0.000	0.000	0.182	0.000	0.000	0.000	0.000	0.091	0.091	0.000	0.045	0.000	0.000
Jinhua	14	0.071	0.000	0.000	0.000	0.286	0.000	0.000	0.000	0.000	0.000	0.000	0.000	0.429	0.000
Laiwu	10	0.000	0.200	0.200	0.000	0.000	0.000	0.000	0.000	0.000	0.300	0.000	0.000	0.000	0.000
Rongchang	4	0.000	0.000	0.000	0.000	0.250	0.000	0.000	0.250	0.000	0.000	0.250	0.000	0.000	0.000
Shaziling	6	0.000	0.000	0.000	0.000	0.167	0.000	0.000	0.167	0.000	0.000	0.167	0.167	0.000	0.000
Tibet	8	0.375	0.000	0.000	0.000	0.125	0.000	0.000	0.000	0.000	0.000	0.000	0.125	0.000	0.000
Tongcheng	10	0.400	0.100	0.000	0.100	0.100	0.000	0.200	0.100	0.000	0.000	0.000	0.000	0.000	0.000
Yushan Black	26	0.462	0.000	0.000	0.154	0.231	0.000	0.000	0.000	0.000	0.000	0.000	0.038	0.000	0.000
Western breeds	102	0.000	0.373	0.284	0.000	0.000	0.118	0.000	0.000	0.059	0.000	0.000	0.000	0.000	0.059
Duroc	14	0.000	0.071	0.071	0.000	0.000	0.071	0.000	0.000	0.000	0.000	0.000	0.000	0.000	0.357
Landrace	38	0.000	0.474	0.289	0.000	0.000	0.211	0.000	0.000	0.026	0.000	0.000	0.000	0.000	0.000
Large White	50	0.000	0.380	0.340	0.000	0.000	0.060	0.000	0.000	0.100	0.000	0.000	0.000	0.000	0.020

N, Number of haplotypes, the 14 haplotypes were ordered by their frequencies in all tested animals. Red (blue) background represents the haplotypes that are unique to Chinese (Western) breeds.

**Figure 1:**
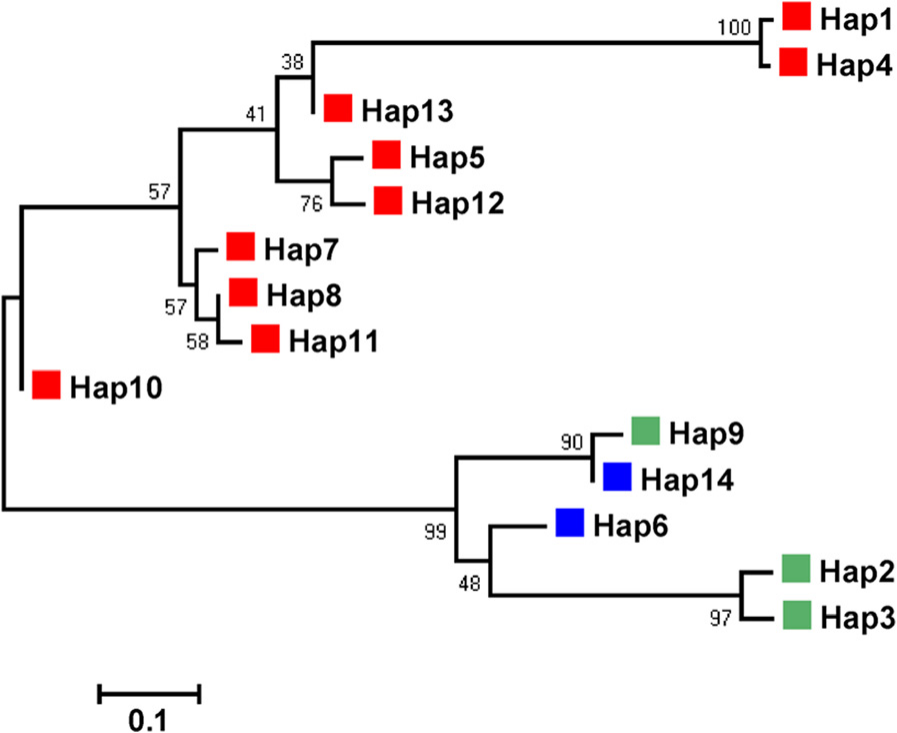
**Neighbor-Joining tree of 14 *MUC4 *major haplotypes with frequencies large than 0.02.** Haplotypes specific for Chinese indigenous breeds and Western commercial breeds are indicated in red and blue, respectively. Haplotypes presented in both Chinese and Western breeds are highlighted in green.

**Figure 2:**
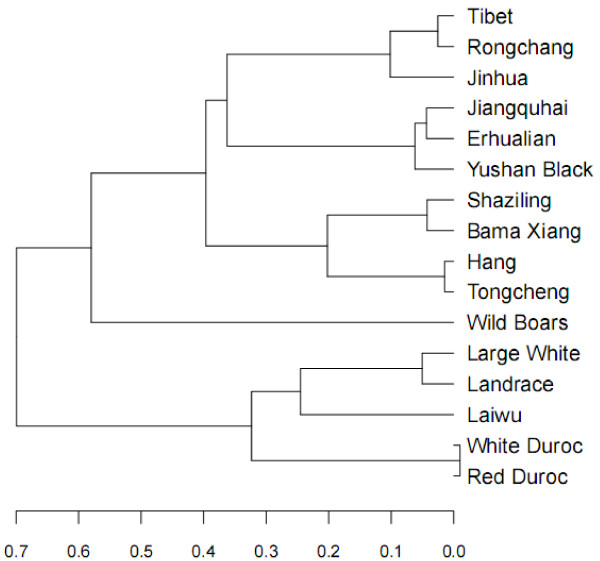
**Dendrogram of the tested breeds based on pairwise breed F**_**ST **_**values.**

We constructed a *MUC4* haplotype phylogenic tree, in which all Chinese haplotypes and Western haplotypes were clustered separately into distinct clades (Figure 1). This suggested that the *MUC4* gene has undergone divergent evolution in Chinese and Western breeds. The divergent evolution pattern reflected independent domestication centers of modern pig breeds across Eurasia [23]. We also calculated breed-pairwise F_ST_ values (Additional file 4: Table S3) using DnaSP V5.10, and constructed a UPGMA dendrogram of the tested breeds by treating the F_ST_ values as distance between breeds. Again, except for Laiwu pigs, Chinese indigenous breeds and Western commercial breeds were grouped into separate clades in the dendrogram (Figure 2), supporting divergent evolution of the *MUC4* gene and providing evidence for the notion that Asian and European breeds have experienced different domestication and breed formation histories [16,23,24]. Moreover, Chinese indigenous breeds that have neighboring geographical locations were usually grouped together. For instance, four belted breeds including Tongcheng, Hang, Bama Xiang and Shaziling that pertained to the Central China Type were clustered into the same sub-branch, and two highly prolific breeds of Erhualian and Jiangquhai pigs belonging to the Lower Yangtze River Basin Type were grouped into a clade (Figure 2). This is not unexpected considering that breeds of geographically close origin could more likely share common ancestors or cross to each other.

### Linkage disequilibrium in the MUC4 gene

**Figure 3:**
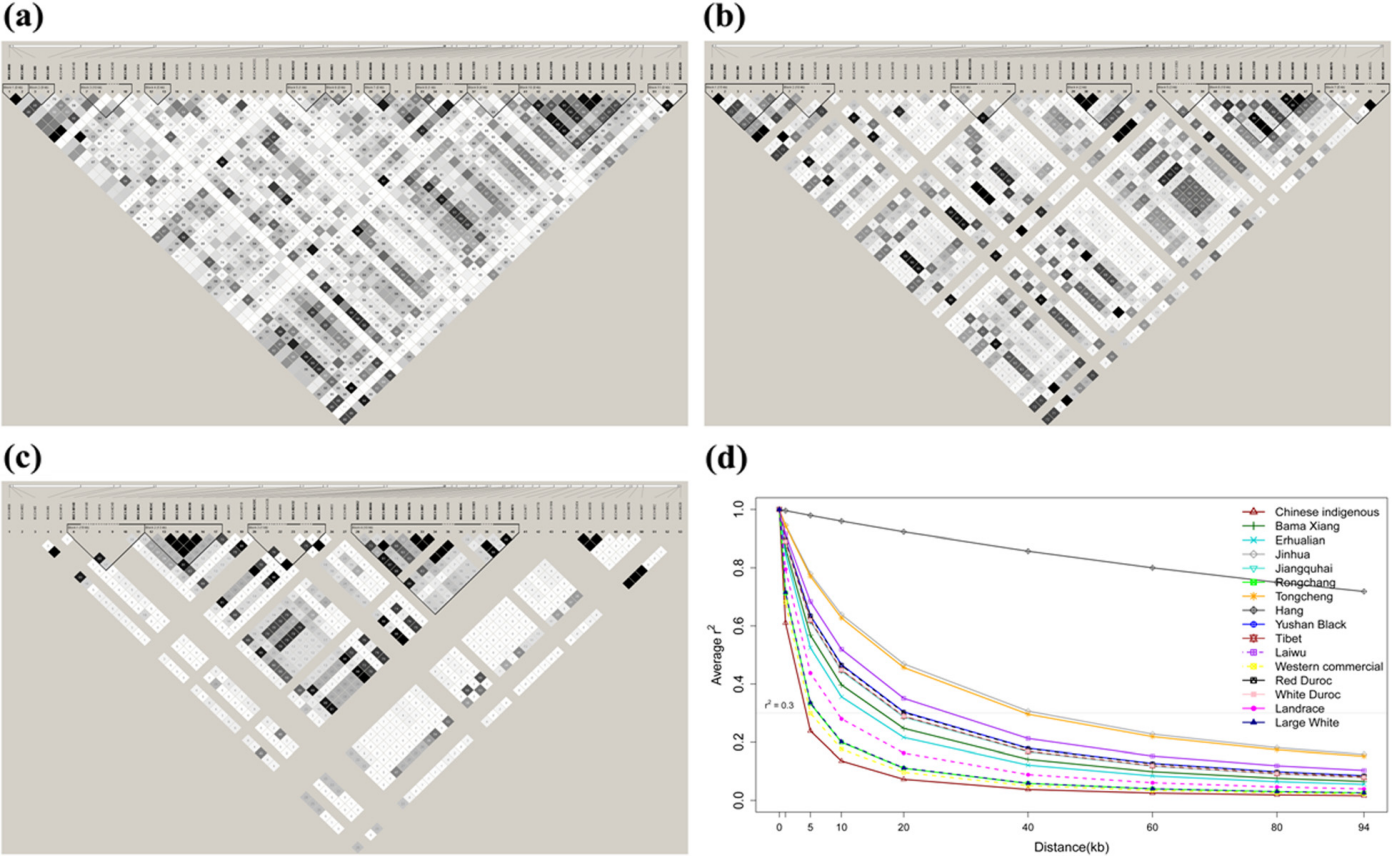
**Haplotype structure and linkage disequilibrium decay of *MUC4 *in the tested breeds.** The *r*^2^ plots between pairs of loci are shown for all animals (**a**), Chinese indigenous breeds (**b**) and Western commercial breed (**c**). LD decay plots are depicted for Chinese and Western breeds as well as individual breeds (**d**).

In the present study, we employed *r*^*2*^ values as measures of LD, as it is less affected by allele frequency and is independent from sample size compared with *D’* values [25]. Using the genotypes of 53 *MUC4* SNPs from all 312 animals, 11 haplotype blocks with an average size of 3.9 kb (ranging from 0.013 to 10 kb) were inferred (Figure 3a). Thirty-five SNPs were required to capture all 53 loci at *r*^2^ ≥ 0.96. We further surveyed haplotype blocks partitioning separately in Chinese and Western breeds. Eight and 4 haplotype blocks were detected in Chinese and Western breeds, respectively (Figure 3b and c). The maximum LD block size was 15 and 19 kb in Chinese and Western breeds respectively. Moreover, 16 and 22 SNPs were not assigned to any haplotype block in Chinese and Western breeds respectively (Figure 3b and c). The low LD extent of *MUC4* in Western breeds is contrast to the previously reported ~400 kb haplotype blocks in three different genomic regions (1~3 cM) in these breeds [20], which might be caused by high recombination and mutation rates within the *MUC4* region. Our observations suggested that higher density markers e.g., 1SNP/10 kb, are required to capture potential functional DNA variants of genes like *MUC4* in the pig genome. In this sense, the current available porcine 60 SNP chip with a marker density of 1SNP/50 kb could lead to false negative result in genome-wide association studies due to insufficient capturing ability of LD markers. Of note, despite having similar haplotype block sizes, the locations of haplotype blocks differ across Chinese and Western breeds (Figure 3b and c). This could result from the divergent evolution histories of Chinese and Western breeds at *MUC4* as suggested by above-mentioned analyses.

We further examined the LD decline against genomic distance in breeds with sample size greater than 10 (Figure 3d). We used the same equation to fit the LD decay as that used by Amaral et al. (2008) to make our result comparable to their results. We assessed the fit goodness of inferred decay using the correlation between the observed and predicted LD measures (*r*^*2*^), which varied from 0.1 to 0.33 in different breeds. With a threshold of r^2^ = 0.3, we observed that the LD extended less than 20 kb in all Western breeds. Large White pigs have the smallest LD extent (~5 kb) followed by Landrace (~10 kb) and Duroc (~20 kb). These results are in stark contrast to those reported by Amaral et al. (2008) [20] where they showed that LD extend a much longer distance. i.e., from 0.1 Mb to 2 Mb, across three genomic regions in Western breeds. Chinese breeds exhibited different patterns of LD decay. Rongchang pigs had the lowest (~7 kb) LD extent, while Hang pigs had the highest LD extent (more than 100 kb). It is known that LD pattern in a population is determined by the domestication and demographic history of the population. The higher LD extent reflects stronger bottleneck effects or smaller effective population size. Therefore, Hang pigs could have much smaller effective population size than other Chinese breeds, such as Rongchang and Erhualian. Analyses of more individuals and genomic regions would give more conclusive evidence for this assumption.

### Balancing selection in the MUC4 gene?

In this study, the Tajima’D, Fu and Li’s D statistics were used to test whether *MUC4* evolved neutrally or undergo directional or balancing selection. Directional selection usually results in negative values of these statistics while balancing selection causes positive values. We detected significant positive Tajima’ D, or Fu and Li’s D statistics in both Chinese and Western breeds. Especially, all Chinese breeds except Erhualian and Jinhua and all Western breeds except Landrace had significant positive Fu and Li’s D statistics in the *MUC4* region (Table 1). We speculated that the observations were less likely caused by demographic effects, provided that the same individuals from Rongchang, Tongcheng, Duroc and Large White breeds were also tested for their D statistics in the *PPARD* gene, and no significant positive D values were observed in these pig populations [4]. However, we can’t exclude the possibility that the observed significant positive D statistics were indeed upward biased due to SNP acertainment bias, given that *MUC4* was not fully sequenced and the tested SNPs were identified using only two divergent breeds. In all, our analysis supported the hypothesis that *MUC4* could have undergone balancing selection in both Chinese and Western breeds. The hypothesis is also favored by the above-mentioned finding of the high nucleotide variability of *MUC4* in Chinese and Western breeds (Table 2). Maintaining high nucleotide diversity could facilitate multiple biological functions fulfilled by the *MUC4* gene [8], therefore beneficial to the fitness of individuals.

## Conclusion

Both Chinese and Western breeds have considerable genetic variability within the *MUC4* gene. Linkage disequilibrium at this gene is similar between Chinese and Western pig breeds, normally extending less than ~20 kb. Moreover, Chinese and Western breeds have evolved divergently but both could have undergone balancing selection at the *MUC4* locus.

## Methods

### Animals

Experimental animals included 307 unrelated pigs with no common ancestry for 3 generations, 4 Chinese wild boars and 1 European wild boar. The 307 animals pertained to 11 Chinese indigenous breeds including Bama Xiang, Erhualian, Hang, Jinhua, Jiangquhai, Laiwu, Rongchang, Shaziling, Tongcheng, Yushan Black and Tibet, and 3 Western commercial breeds comprising Duroc, Landrace and Large White. Chinese pigs breeds have been classified into 6 ecotypes including North China, South China, Central China, Southwest China, Lower Yangtze River Basin and Plateau types according to their appearance, geological location and performance [26]. The 11 Chinese breeds used in this study represent all 6 ecotypes (Table 1). Genomic DNA was extracted from ear tissues using a standard phenol/chloroform method and was diluted to a final concentration of 20 ng/μl in 96-well plates.

### SNP identification and genotyping

To identify *MUC4* SNPs, we designed 32 primer pairs (Additional file 1: Table S1) to amplify fragments of 121-1800 bp that span a 92-kb region around *MUC4*. All primer pairs were designed using the Primer5 software ( http://www.premierbiosoft.com/). Template DNA was extracted from four White Duroc pigs and four Chinese Erhualian pigs. Amplifications were performed in a 25-μl final volume containing 1.5 mM MgCl_2_, 0.5 mM dNTPs, 0.5 μM of each primer, 40 ng DNA, and 2.5 unit Taq DNA polymerase (Bocai, Shanghai, China). Thermocycling parameters were 95°for 5 min, 35 cycles of 95°for 30 s, optimal annealing temperatures (Additional file 1: Table S1) for 30 s, 72°for 40 s, with a final extension of 72°for 10 min. PCR products were purified with the QIAquick DNA Purification kit (Qiagen, Hilden, Germany) and sequenced with the respective PCR primers by a 3130XL Genetic Analyzer (ABI, Foster City, U.S.A). The identities of amplicons were checked using the blastn program via the NCBI BLAST server (http:// http://www.ncbi.nlm.nih.gov/BLAST/). SNPs were identified through manual checking and verification after aligning the obtained sequences by using the DNAstar software ( http://www.dnastar.com/). Highly polymorphic SNPs were chosen for further genotyping the above-mentioned 312 animals by using the MassARRAY SNP genotyping system (Sequenom, San Diego, U.S.A) or the ABI Snapshot protocol (ABI, Foster City, U.S.A). The MassARRAY assay combined a reproducible primer extension reaction chemistry with MALDI-TOF mass spectrometry to quickly characterize genotypes. The SNaPshot technology allowed for the detection of up to 10 know SNPs in a single run on 3130XL Genetic Anayzer (ABI, Foster City, U.S.A) by using varied length of primer and incorporating a fluorescent labeled dideoxynucleotide at desired SNP site. SNPs passing the filtering criteria with call rates >90% were used for further analyses.

### Data analysis

Haplotype phases were inferred with Phase v2.1.1 [27]. The program was performed with 1000 iterations, and the last iteration was 10 times longer than the default setting as suggested by the developers. All inferred haplotypes were used to determine the following statistics using DnaSP v5.10 [28]: the number of segregating site (S), the mean number of pairwise differences across loci (π_N_), Tajima’s D, Fu and Li’s D, and the measure of population differentiation (F_ST_) between breed pairs. Only those haplotypes with probabilities of more than 0.8 (*P* > 0.8) were used for phylogenic and molecular evolutionary analyses. Neighbor-Joining haplotype tree was constructed using MEGA v5 [29]. The haplotype blocks and the plots of linkage disequilibrium (*r*^*2*^) were created with Haploview V4.2 [30]. The dendrogram of the tested breeds based on breed pairwise F_ST_ was drawn using *hclust* function in R. The LD decay plot were created using the approach described in [20]. In brief, for each sub-population with more than 10 individuals, all pairwise SNP LD measures (*r*^*2*^) were calculated using R package *genetics*[31]. Then, equation 1 in [20] was fitted using R function *nls* to estimate coefficients that describe the decline of *r*^*2*^ values with distance. Predicted values *r*^*2*^ were plotted against the distances based on the estimates.

## Competing interests

The authors declare that they have no competing interests.

## Author’s contribution

Conceived and designed the experiments: JR and LH. Performed the experiments: MY, XY, JO and WZ. Analyzed the data: BY, MY, HA and JR. Wrote the paper: BY, MY and JR. Provided comments for the manuscript: LH. All authors read and approved the final manuscript.

## Supplementary Material

Additional file 1: Table S1 ** Primers for identification of SNP markers in the region of *MUC4 *gene that were genotyped in outbred populations.** Click here for file

Additional file 2: Figure S1 ** The genomic structure of the porcine *MUC4 *gene (lower panel) and locations of 53 SNPs covering a 92-kb region around *MUC4 *(upper and lower panels).** Blue boxes indicate exons and thin lines indicate introns. Untranslated regions at 5’ and 3’ end are highlighted in yellow. Exon 2 of *MUC4* is a tandem repetitive region in which no SNP was genotyped in this study. Click here for file

Additional file 3: Table S2 ** Minor allele frequencies and heterozygosities of 90 SNPs identified by sequencing 4 Duroc and 4 Erhualian pigs.** The 53 SNPs that were genotyped in the 312 pigs were highlighted in red colors. Click here for file

Additional file 4: Table S3 The measures of fixation index (lower triangle) and average number of pairwise nucleotide differences (upper triangle) between pairs of breeds. Click here for file
