# Supplementary material for: Nucleotide variability and linkage disequilibrium patterns in the porcine MUC4 gene
Source: BMC Genet. 2012 Jul 13;13:57. doi: 10.1186/1471-2156-13-57 (PMC3505144; doi:10.1186/1471-2156-13-57)
Supplement: Additional file 1: Table S1 — Primers for identification of SNP markers in the region of MUC4 gene that were genotyped in outbred populations. [file 1471-2156-13-57-S1.doc]

**Supplementary Table 1.** Primers for identification of SNP markers in the region of *MUC4* gene that were genotyped in outbred populations.

| No. | Forwardprimer  (5’-3’) | Reverseprimer  (5’-3’) | Tm  (^o^C) | Amplicon(bp) | Gene | SNP | Pos.onSSC13  (Sscrofa9.2,bp) |
| --- | --- | --- | --- | --- | --- | --- | --- |
|  | GCTCACCTATTCCCACAAACG | CCTCTTCATAGGGGATGT | 60 | 1288 | *MUC4-M0B* | A>C | 100970506 |
|  | GCTCACCTATTCCCACAAACG | CCTCTTCATAGGGGATGT | 60 | 1288 | *MUC4-M0C* | G>A | 100970692 |
|  | GCTCACCTATTCCCACAAACG | CCTCTTCATAGGGGATGT | 60 | 1288 | *MUC4-M0* | T>C | 100971008 |
|  | GGGATTAGGGGAAACCTTGGC | TCCCAACCCTGAAACCACCTC | 63 | 1404 | *MUC4-M9* | T>C | 100980382 |
|  | CAGTTGGGCAGAATGTTTATGAC | GCACGCTGATGCTACTACCG | 59 | 1394 | *MUC4-M14* | C>T | 100984911 |
|  | CAGTTGGGCAGAATGTTTATGAC | GCACGCTGATGCTACTACCG | 59 | 1394 | *MUC4-M14B* | T>A | 100985776 |
|  | CCAGAAGCAGATAAGTGAGAAC | GATACCAGATGTGGAAGGATG | 54 | 1315 | *MUC4-M19B* | C>T | 100990431 |
|  | CCAGAAGCAGATAAGTGAGAAC | GATACCAGATGTGGAAGGATG | 54 | 1315 | *MUC4-M19* | A>T | 100990686 |
|  | AATGCCTCCTGGATTACTTTC | CTGGAATCTAATACACGGCAC | 55 | 1345 | *MUC4-M24B* | A>C | 100996185 |
|  | CAGCAAGAGCCCTGACAAGCC | GCTGAAGGGAACTGAGGTGGATAG | 64 | 1380 | *MUC4-M24* | C>T | 101000742 |
|  | CCAGCAAAGACTTGGCACCTAC | GCAAATACGGATGCCCAGTTC | 62 | 1422 | *MUC4-M34* | A>G | 101004962 |
|  | CCAGCAAAGACTTGGCACCTAC | GCAAATACGGATGCCCAGTTC | 62 | 1422 | *MUC4-M34C* | G>A | 101005376 |
|  | CACAGCGGTAGTGAAGAAATC | CTGCTTCTATCTTCTGGCTCTATG | 59 | 1422 | *MUC4-M39B* | G>A | 101010597 |
|  | CACAGCGGTAGTGAAGAAATC | CTGCTTCTATCTTCTGGCTCTATG | 59 | 1422 | *MUC4-M39* | T>A | 101010998 |
|  | CAACGGAGAGGGTATTGGGAAGC | AGCCTACGCCAGAGCCACAGC | 66 | 1117 | *MUC4-M43B* | A>G | 101013757 |
|  | CAACGGAGAGGGTATTGGGAAGC | AGCCTACGCCAGAGCCACAGC | 66 | 1117 | *MUC4-M43* | T>C | 101014346 |
|  | GCTTAGACAGTGAGACATCAACATC | AATTACAGGTGACACGCTTCC | 57 | 1366 | *MUC4-M47* | C>T | 101018667 |
|  | TTCCAGGAGTTTGATTGTGTC | GTCATAGTGTTTCCACCTGTAGAG | 55 | 1159 | *MUC4-M51* | A>G | 101022151 |
|  | TTCCAGGAGTTTGATTGTGTC | GTCATAGTGTTTCCACCTGTAGAG | 55 | 1159 | *MUC4-M51B* | A>C | 101022561 |
|  | CAGAGAAACCCACAGCAGCG | GGGTAAAGAGGATGGTGAGGC | 62 | 1800 | *MUC4-M2432C* | T>C | 101030382 |
|  | CAGAGAAACCCACAGCAGCG | GGGTAAAGAGGATGGTGAGGC | 62 | 1800 | *MUC4-M2432B* | C>G | 101030400 |
|  | ACCCTCTTCCACCACCTCTG | GAATGTCCTCTCTCAAGCACTC | 61 | 508 | *MUC4-M60* | C>T | 101030548 |
|  | CAGAGAAACCCACAGCAGCG | GGGTAAAGAGGATGGTGAGGC | 62 | 1800 | *MUC4-M2432* | G>C | 101030595 |
|  | GACATCATCAGTGTTGGGTGC | TCAATCACAGTCACAGTTATGGC | 58 | 1339 | *MUC4-M61B* | A>G | 101031462 |
|  | GACATCATCAGTGTTGGGTGC | TCAATCACAGTCACAGTTATGGC | 58 | 1339 | *MUC4-M61* | G>A | 101032154 |
|  | AAAAGGGTGACTCAGCAATGG | ACAAGAAGGCAGATTCAGACAAG | 59 | 1295 | *MUC4-M63* | A>G | 101033834 |
|  | CCAGCAAGGACAGACAGAGG | CCACCTGTGCCCATCTGTATC | 58 | 420 | *MUC4-M64* | C>T | 101033847 |
|  | TTGTCACTGCTGTGGCTCTG | CCTGGCTATTTTCTTTCTATTTC | 56 | 121 | *MUC4-M6862* | T>C | 101034830 |
|  | TTCACTCTGCCGTTCTCTTTC | CAGCTGTTCAGTCGTTTCGTC | 60 | 1161 | *MUC4-M66B* | C>G | 101036195 |
|  | TTCACTCTGCCGTTCTCTTTC | CAGCTGTTCAGTCGTTTCGTC | 60 | 1161 | *MUC4-M66C* | G>A | 101036462 |
|  | TTCACTCTGCCGTTCTCTTTC | CAGCTGTTCAGTCGTTTCGTC | 60 | 1161 | *MUC4-M66* | G>A | 101036804 |
|  | GACATCTATTCAGTCTTCGTTTATTG | AAGTCCCATAGTCCCTGCTGC | 59 | 1188 | *MUC4-M67B* | A>G | 101038525 |
|  | GACATCTATTCAGTCTTCGTTTATTG | AAGTCCCATAGTCCCTGCTGC | 59 | 1188 | *MUC4-M67* | T>G | 101038734 |
|  | TGTCCACAGAGAACTTGAGCC | GTTTGCCATCTCCACACACTG | 58 | 1306 | *MUC4-M69* | C>T | 101039711 |
|  | TGTCCACAGAGAACTTGAGCC | GTTTGCCATCTCCACACACTG | 58 | 1306 | *MUC4-M69B* | T>C | 101039962 |
|  | TGTCCACAGAGAACTTGAGCC | GTTTGCCATCTCCACACACTG | 58 | 1306 | *MUC4-M69C* | A>G | 101040542 |
|  | GAAACATAGGATTAGGGTCTTGG | TGGATTGTGTCATTGGGCTC | 58 | 354 | *MUC4 -13383* | C>T | 101041357 |
|  | CTCTCAGCCTCCTCTCCACATC | AAGTCATCATCTGGGTTGCCG | 61 | 1365 | *MUC4-M71* | G>C | 101042640 |
|  | CAGGATGCCCAATGGCTCTAC | CCCCGAAGTTGTGAAAGGAAG | 65 | 538 | *MUC4-16100* | A>G | 101043569 |
|  | GTGCCTTTCTTGGCTAACTTGTC | TGTGGCTGTGGTGTAGGTTGG | 61 | 1218 | *MUC4-M74* | C>T | 101045566 |
|  | TGGGACGTTGCTATGGACACC | CTCTACTGAGACGGCTCTGGAAAG | 62 | 1447 | *MUC4-M77* | T>C | 101047460 |
|  | TGGGACGTTGCTATGGACACC | CTCTACTGAGACGGCTCTGGAAAG | 62 | 1447 | *MUC4-M77B* | G>A | 101047871 |
|  | CAGTAGAGGAGTTAGAGAGACCCG | CAGAGCCACAGCAACGCAG | 61 | 1348 | *MUC4-21569* | C>T | 101049579 |
|  | CCTTGGCTAAGTCTGGGTAAAC | AAATGAACAGCCCCTACCTTG | 58 | 1094 | *MUC4-M81* | C>T | 101051344 |
|  | GTCCTTACCTACACCCCTCAC | TGCCTCCCTACCACACATACC | 60 | 1109 | *MUC4-25454* | T>G | 101053466 |
|  | GTGTAGTCACTGTAGAGGCTTAGG | CTATCCATTTGCTGGTTTGAG | 55 | 1432 | *MUC4-M85B* | A>G | 101055283 |
|  | GTGTAGTCACTGTAGAGGCTTAGG | CTATCCATTTGCTGGTTTGAG | 55 | 1432 | *MUC4- M85C* | G>A | 101056048 |
|  | GTGTAGTCACTGTAGAGGCTTAGG | CTATCCATTTGCTGGTTTGAG | 55 | 1432 | *MUC4-M85* | T>C | 101056292 |
|  | GCCCTACTACAGTCGCAGATG | GGGGTTACTGGGAGATGGTTG | 59 | 1326 | *MUC4 -M87B* | G>A | 101057025 |
|  | GCCCTACTACAGTCGCAGATG | GGGGTTACTGGGAGATGGTTG | 59 | 1326 | *MUC4-M87* | G>A | 101057831 |
|  | GCCCATTATCTAAGTCATTCAGC | AGTATCCTCCCAGCAGGTCTC | 58 | 1334 | *MUC4-M92* | C>T | 101062625 |
|  | GCCCATTATCTAAGTCATTCAGC | AGTATCCTCCCAGCAGGTCTC | 58 | 1334 | *MUC4-M92C* | C>G | 101062791 |
|  | GCCCATTATCTAAGTCATTCAGC | AGTATCCTCCCAGCAGGTCTC | 58 | 1334 | *MUC4-M92B* | G>A | 101062954 |

^a^SeventeenSNPsthatcannotmaptothepiggenomeassembly(Sscrofa9.2)areindicatedby‘-’;

^b^TheseSNPsarelocatedoutsidethe3.1-Mbcriticalregionharboringthereceptorlocus;

^c^SNPsfromJacobsenetal.(2010).
