# Supplementary material for: Nucleotide variability and linkage disequilibrium patterns in the porcine MUC4 gene
Source: BMC Genet. 2012 Jul 13;13:57. doi: 10.1186/1471-2156-13-57 (PMC3505144; doi:10.1186/1471-2156-13-57)
Supplement: Additional file 2: Figure S1 — The genomic structure of the porcine MUC4 gene (lower panel) and locations of 53 SNPs covering a 92-kb region around MUC4 (upper and lower panels). Blue boxes indicate exons and thin lines indicate introns. Untranslated regions at 5’ and 3’ end are highlighted in yellow. Exon 2 of MUC4 is a tandem repetitive region in which no SNP was genotyped in this study. [file 1471-2156-13-57-S2.doc]

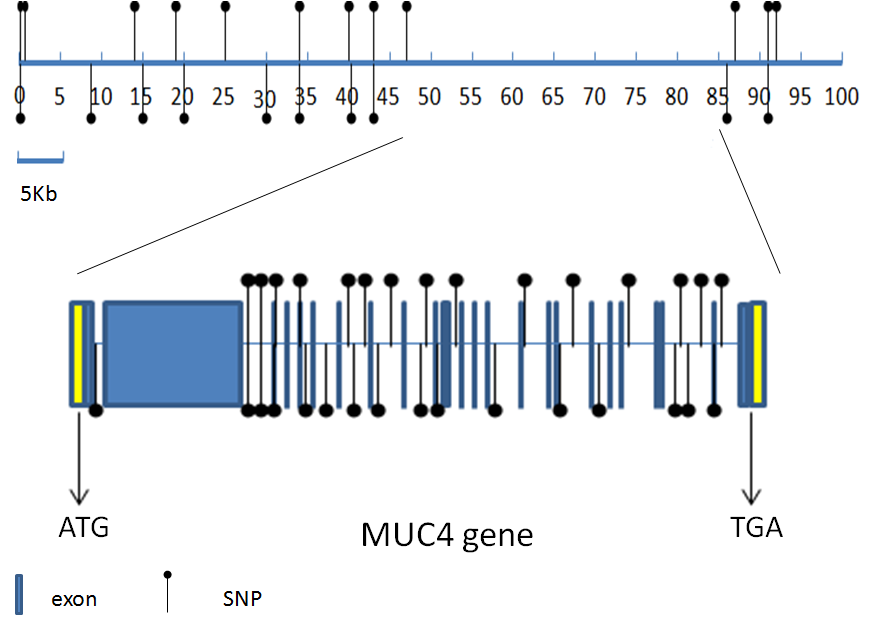


**Supplemental Figure 1**. The genomic structure of the porcine *MUC4* gene (lower panel) and locations of 53 SNPs covering a 92-kb region around *MUC4* (upper and lower panels). Blue boxes indicate exons and thin lines indicate introns. Untranslated regions at 5’ and 3’ end are highlighted in yellow. Exon 2 of *MUC4* is a tandem repetitive region in which no SNP was genotyped in this study.
