# Supplementary material for: Nucleotide variability and linkage disequilibrium patterns in the porcine MUC4 gene
Source: BMC Genet. 2012 Jul 13;13:57. doi: 10.1186/1471-2156-13-57 (PMC3505144; doi:10.1186/1471-2156-13-57)
Supplement: Additional file 3: Table S2 — Minor allele frequencies and heterozygosities of 90 SNPs identified by sequencing 4 Duroc and 4 Erhualian pigs. The 53 SNPs that were genotyped in the 312 pigs were highlighted in red colors. [file 1471-2156-13-57-S3.doc]

**Supplemental Table 2**. Minor allele frequencies and heterozygosities of 90 SNPs identified by sequencing 4 Duroc and 4 Erhualian pigs. The 53 SNPs that were genotyped in the 312 pigs were highlighted in red colors.

| SNPs | 4 Duroc | | 4 Erhualian | |
| --- | --- | --- | --- | --- |
|  | MAF | Heterozygosity | MAF | Heterozygosity |
| M0-107 | 0.125 | 0.250 | 0.000 | 0.000 |
| MUC4-M0B | 0.000 | 0.000 | 0.250 | 0.429 |
| MUC4-M0C | 0.000 | 0.000 | 0.250 | 0.429 |
| M0-553 | 0.125 | 0.250 | 0.000 | 0.000 |
| M0-629 | 0.125 | 0.250 | 0.000 | 0.000 |
| MUC4-M0 | 0.125 | 0.250 | 0.125 | 0.250 |
| M0-917 | 0.125 | 0.250 | 0.000 | 0.000 |
| M9-562 | 0.250 | 0.429 | 0.000 | 0.000 |
| M14-78 | 0.250 | 0.429 | 0.125 | 0.250 |
| MUC4-M9 | 0.250 | 0.429 | 0.250 | 0.429 |
| M14-338 | 0.125 | 0.250 | 0.250 | 0.429 |
| MUC4-M14 | 0.333 | 0.533 | 0.250 | 0.429 |
| M19-955 | 0.000 | 0.000 | 0.125 | 0.250 |
| MUC4-M14B | 0.000 | 0.000 | 0.250 | 0.429 |
| MUC4-M19B | 0.000 | 0.000 | 0.250 | 0.429 |
| M24-159 | 0.250 | 0.500 | 0.167 | 0.333 |
| MUC4-M19 | 0.250 | 0.500 | 0.250 | 0.500 |
| M29-25 | 0.250 | 0.429 | 0.500 | 0.600 |
| MUC4-M24B | 0.167 | 0.333 | 0.250 | 0.500 |
| MUC4-M24 | 0.250 | 0.429 | 0.250 | 0.429 |
| MUC4-M34 | 0.375 | 0.536 | 0.125 | 0.250 |
| M34-1119 | 0.000 | 0.000 | 0.167 | 0.333 |
| M39-245 | 0.375 | 0.536 | 0.250 | 0.500 |
| MUC4-M34C | 0.375 | 0.536 | 0.000 | 0.000 |
| M39-581 | 0.375 | 0.536 | 0.000 | 0.000 |
| M39-628 | 0.375 | 0.536 | 0.250 | 0.500 |
| MUC4-M39B | 0.375 | 0.536 | 0.250 | 0.500 |
| MUC4-M39 | 0.375 | 0.536 | 0.125 | 0.250 |
| M43-177 | 0.000 | 0.000 | 0.375 | 0.536 |
| M43-160 | 0.375 | 0.536 | 0.125 | 0.250 |
| MUC4-M43B | 0.375 | 0.536 | 0.125 | 0.250 |
| MUC4-M43 | 0.375 | 0.536 | 0.125 | 0.250 |
| M43-104 | 0.375 | 0.536 | 0.125 | 0.250 |
| MUC4-M47 | 0.375 | 0.536 | 0.125 | 0.250 |
| M45-181 | 0.000 | 0.000 | 0.167 | 0.333 |
| MUC4-M51 | 0.500 | 0.667 | 0.167 | 0.333 |
| MUC4-M64 | 0.500 | 0.667 | 0.167 | 0.333 |
| MUC4-M6862 | 0.500 | 0.667 | 0.250 | 0.429 |
| M49-1405 | 0.500 | 0.667 | 0.333 | 0.533 |
| MUC4-M51B | 0.333 | 0.533 | 0.250 | 0.429 |
| M51-393 | 0.000 | 0.000 | 0.250 | 0.429 |
| MUC4-M2432C | 0.000 | 0.000 | 0.000 | 0.000 |
| M51-523 | 0.333 | 0.533 | 0.000 | 0.000 |
| MUC4-M2432B | 0.250 | 0.429 | 0.250 | 0.429 |
| MUC4-M60 | 0.250 | 0.429 | 0.375 | 0.536 |
| M2432-267 | 0.250 | 0.429 | 0.250 | 0.429 |
| M66B | 0.250 | 0.429 | 0.250 | 0.429 |
| M66C | 0.250 | 0.429 | 0.250 | 0.429 |
| M2432-138 | 0.250 | 0.429 | 0.250 | 0.429 |
| MUC4-M2432 | 0.250 | 0.429 | 0.375 | 0.536 |
| MUC4-M61B | 0.500 | 0.571 | 0.250 | 0.500 |
| MUC4-M61 | 0.125 | 0.250 | 0.125 | 0.250 |
| M63-304 | 0.000 | 0.000 | 0.250 | 0.500 |
| MUC4-M63 | 0.000 | 0.000 | 0.125 | 0.250 |
| M63-784 | 0.000 | 0.000 | 0.125 | 0.250 |
| M66 | 0.333 | 0.533 | 0.125 | 0.250 |
| MUC4-M67B | 0.333 | 0.533 | 0.250 | 0.429 |
| M69-1008 | 0.250 | 0.429 | 0.125 | 0.250 |
| M69-972 | 0.250 | 0.429 | 0.000 | 0.000 |
| MUC4-M67 | 0.250 | 0.429 | 0.125 | 0.250 |
| M69 | 0.250 | 0.429 | 0.125 | 0.250 |
| M69B | 0.250 | 0.429 | 0.125 | 0.250 |
| M69C | 0.375 | 0.536 | 0.000 | 0.000 |
| MUC4-M13383 | 0.000 | 0.000 | 0.250 | 0.429 |
| M74-616 | 0.000 | 0.000 | 0.125 | 0.250 |
| MUC4-M71 | 0.000 | 0.000 | 0.125 | 0.250 |
| MUC4-M16100 | 0.000 | 0.000 | 0.250 | 0.429 |
| M77-615 | 0.000 | 0.000 | 0.250 | 0.429 |
| M77-708 | 0.000 | 0.000 | 0.125 | 0.250 |
| MUC4-M74 | 0.375 | 0.536 | 0.000 | 0.000 |
| MUC4-M77 | 0.000 | 0.000 | 0.000 | 0.000 |
| M85-557 | 0.000 | 0.000 | 0.125 | 0.250 |
| MUC4-M77B | 0.000 | 0.000 | 0.125 | 0.250 |
| M85-476 | 0.000 | 0.000 | 0.125 | 0.250 |
| MUC4-21569 | 0.000 | 0.000 | 0.125 | 0.250 |
| M85-92 | 0.500 | 0.571 | 0.125 | 0.250 |
| MUC4-M81 | 0.000 | 0.000 | 0.125 | 0.250 |
| M87-472 | 0.000 | 0.000 | 0.167 | 0.333 |
| MUC4-25454 | 0.000 | 0.000 | 0.167 | 0.333 |
| MUC4-M85B | 0.375 | 0.536 | 0.000 | 0.000 |
| MUC4-M85C | 0.000 | 0.000 | 0.250 | 0.429 |
| MUC4-M85 | 0.000 | 0.000 | 0.250 | 0.429 |
| M92-642 | 0.000 | 0.000 | 0.250 | 0.429 |
| MUC4-M87B | 0.000 | 0.000 | 0.250 | 0.429 |
| MUC4-M87 | 0.375 | 0.536 | 0.125 | 0.250 |
| M8227-306 | 0.000 | 0.000 | 0.250 | 0.429 |
| MUC4-M92 | 0.375 | 0.536 | 0.125 | 0.250 |
| MUC4-M92C | 0.375 | 0.536 | 0.250 | 0.429 |
| M8227-478 | 0.375 | 0.536 | 0.125 | 0.250 |
| MUC4-M92B | 0.250 | 0.429 | 0.125 | 0.250 |
