# Supplementary material for: Nucleotide variability and linkage disequilibrium patterns in the porcine MUC4 gene
Source: BMC Genet. 2012 Jul 13;13:57. doi: 10.1186/1471-2156-13-57 (PMC3505144; doi:10.1186/1471-2156-13-57)
Supplement: Additional file 4: Table S3 — The measures of fixation index (lower triangle) and average number of pairwise nucleotide differences (upper triangle) between pairs of breeds. [file 1471-2156-13-57-S4.doc]

Supplemental Table 3. The measures of fixation index (lower triangle) and average number of pairwise nucleotide differences (upper triangle) between pairs of breeds.

|  | Bama Xiang | Erhualian | Jinhua | Jiangquhai | Rongchang | Laiwu | Shaziling | Tongcheng | Hang | Yushan Black | Tibet | Red Duroc | Landrace | Large White | White Duroc | Wild Boars |
| --- | --- | --- | --- | --- | --- | --- | --- | --- | --- | --- | --- | --- | --- | --- | --- | --- |
| Bama Xiang | 0.000 | 18.732 | 14.551 | 14.529 | 16.000 | 21.192 | 13.169 | 12.263 | 21.848 | 10.061 | 15.083 | 12.630 | 20.788 | 12.953 | 21.488 | 26.038 |
| Erhualian | 0.378 | 0.000 | 17.005 | 16.894 | 13.359 | 21.001 | 12.561 | 11.082 | 13.242 | 25.639 | 12.913 | 14.915 | 20.708 | 21.674 | 11.875 | 26.823 |
| Jinhua | 0.312 | 0.363 | 0.000 | 12.387 | 17.117 | 21.366 | 14.072 | 14.194 | 9.986 | 25.631 | 13.002 | 12.619 | 22.029 | 22.646 | 12.321 | 26.831 |
| Jiangquhai | 0.199 | 0.044 | 0.217 | 0.000 | 15.169 | 21.176 | 23.661 | 30.298 | 17.192 | 25.921 | 14.406 | 12.364 | 19.224 | 22.728 | 26.258 | 26.185 |
| Rongchang | 0.145 | 0.232 | 0.083 | 0.081 | 0.000 | 20.477 | 16.973 | 30.685 | 11.574 | 25.824 | 26.878 | 11.747 | 18.819 | 21.938 | 28.088 | 18.740 |
| Laiwu | 0.196 | 0.494 | 0.400 | 0.341 | 0.295 | 0.000 | 14.300 | 30.903 | 14.527 | 19.795 | 27.579 | 25.523 | 14.951 | 19.516 | 27.794 | 14.316 |
| Shaziling | 0.043 | 0.396 | 0.257 | 0.206 | 0.128 | 0.249 | 0.000 | 30.317 | 12.250 | 13.907 | 27.743 | 25.486 | 16.411 | 12.315 | 26.272 | 13.307 |
| Tongcheng | 0.098 | 0.103 | 0.231 | 0.019 | 0.089 | 0.290 | 0.140 | 0.000 | 10.750 | 21.147 | 27.130 | 25.812 | 19.099 | 14.219 | 20.500 | 11.754 |
| Hang | 0.179 | 0.237 | 0.371 | 0.123 | 0.198 | 0.455 | 0.202 | 0.014 | 0.000 | 15.391 | 21.208 | 25.635 | 22.808 | 14.913 | 12.095 | 23.832 |
| Yushan Black | 0.298 | 0.062 | 0.186 | 0.049 | 0.097 | 0.440 | 0.291 | 0.073 | 0.182 | 0.000 | 18.053 | 20.404 | 15.156 | 26.011 | 29.137 | 10.713 |
| Tibet | 0.189 | 0.247 | 0.102 | 0.121 | 0.025 | 0.344 | 0.159 | 0.097 | 0.188 | 0.087 | 0.000 | 16.212 | 11.981 | 26.053 | 29.753 | 14.389 |
| Red Duroc | 0.414 | 0.643 | 0.643 | 0.525 | 0.547 | 0.323 | 0.492 | 0.478 | 0.615 | 0.617 | 0.560 | 0.000 | 14.948 | 26.314 | 29.864 | 25.156 |
| Landrace | 0.478 | 0.695 | 0.699 | 0.589 | 0.603 | 0.245 | 0.578 | 0.534 | 0.691 | 0.674 | 0.627 | 0.293 | 0.000 | 25.944 | 29.164 | 13.409 |
| Large White | 0.416 | 0.649 | 0.645 | 0.538 | 0.550 | 0.221 | 0.513 | 0.482 | 0.634 | 0.625 | 0.571 | 0.127 | 0.051 | 0.000 | 20.958 | 24.869 |
| White Duroc | 0.399 | 0.634 | 0.634 | 0.518 | 0.537 | 0.317 | 0.484 | 0.465 | 0.603 | 0.607 | 0.551 | -0.010 | 0.276 | 0.111 | 0.000 | 23.742 |
| Wild Boars | 0.435 | 0.544 | 0.580 | 0.437 | 0.474 | 0.478 | 0.479 | 0.407 | 0.547 | 0.508 | 0.434 | 0.550 | 0.631 | 0.566 | 0.535 | 0.000 |
